# Isolated Right Ventricular Metastasis of Hepatocellular Carcinoma: Clinical Findings and Histopathology of an Atypical Presentation

**DOI:** 10.1002/jgh3.70079

**Published:** 2024-12-20

**Authors:** Aaron Yee Shuen See, Pulkit Kasiviswanathan, Masood Pasha Syed, Marta Minervini, Ibrahim Halil Sahin, Michal Krauze

**Affiliations:** ^1^ Medical School University of Western Australia Perth Western Australia Australia; ^2^ Hamdard Institute of Medical Sciences and Research New Delhi India; ^3^ Hillman Cancer Center University of Pittsburgh Medical Center Pittsburgh Pennsylvania USA; ^4^ Division of Transplantation Pathology University of Pittsburgh Medical Center Pittsburgh Pennsylvania USA

**Keywords:** hepatocellular carcinoma, metastasis, polycythemia, Wnt beta‐catenin signaling pathway

## Abstract

Cardiac metastases are a rare site for metastatic hepatocellular carcinoma (HCC). We describe an atypical presentation of an isolated right ventricular metastasis of HCC following successful treatment with no evidence of primary disease recurrence. The case presented as gradually worsening hypertension and erythrocytosis in the setting of normal surveillance scans and alpha‐fetoprotein levels. The mass was detected on transthoracic echocardiogram and treated with surgical resection. Histopathological features of the tumor demonstrated features associated with WNT/β‐catenin mutated HCC, such as microtrabecular, acinar, and bile staining with positive glutamine synthetase expression.

## 1 Introduction

Hepatocellular carcinoma (HCC) is the most common primary tumor of the liver, often associated with conditions such as cirrhosis, chronic hepatitis B and C infection, and alcohol use [1]. Prognosis remains poor for HCC, with a five‐year survival rate of 19.6% [1]. Metastatic disease is common and often found in the lungs, lymph nodes, bones, and adrenal glands [2]. The heart is an uncommon site for metastases, often from primary tumor extension into the right atrium via the inferior vena cava and hepatic vein [3]. Isolated right ventricular (RV) metastasis in the absence of contiguous spread and primary recurrence represents an even rarer occurrence [3, 4]. Here we present a case of isolated right RV metastasis that manifested with erythrocytosis and hypertension, which was managed by RV mass resection and tricuspid valve replacement.

## 2 Case Report

The patient is a 66‐year‐old male with a past medical history of resected HCC, primary essential hypertension, and dyslipidemia. The patient was diagnosed with HCC 3 years prior when he complained of right upper quadrant discomfort and weight loss. Computed tomography (CT) scan of the abdomen showed two large right hepatic lobe masses measuring 14.5 and 9.8 cm. No evidence of cirrhosis, portal hypertension, or extrahepatic involvement was noted. Tests for HBV, HCV, and autoimmune liver disease were negative. Subsequent biopsy demonstrated well differentiated HCC with background steatosis, consistent with metabolic dysfunction‐associated fatty liver disease. No evidence of liver inflammation or fibrosis was present. Laboratory studies at the time were remarkable for a raised alkaline phosphatase of 402 IU/L. Other laboratory indices including complete blood count, basic metabolic panel, liver function tests, iron studies, HbA1C, and alpha fetoprotein (AFP) were normal. The patient subsequently underwent portal vein embolization, trans‐arterial chemoembolization with cisplatin, and extended right lobe resection (stage pT3a N0M0) with no residual disease. He had since been followed up every 6 months, with latest surveillance CT scans of the abdomen and pelvis, demonstrating no evidence of disease recurrence along with normal AFP levels.

At the time of presentation, the patient was managed by his primary care physician for refractory hypertension and erythrocytosis during routine follow‐up. For the past few months, the patient noted increasing blood pressure on home measurements, with systolic readings exceeding 160 mmHg, despite aggressive titration of antihypertensives. In addition, it was also noted that he had a gradual increase in hemoglobin (Hb) from baseline 12 g/dL 2 years ago, 14.2 g/dL slightly over a year ago, and 17.4 g/dL on presentation. No erythrocytosis was noted during the initial diagnosis of HCC 3 years ago. However, the patient reported being a frequent blood donor prior to his diagnosis. He was otherwise well, and denied any symptoms such as chest pain, shortness of breath, dizziness, blurry vision, nausea, or significant headache. Physical examination findings, including cardiovascular and respiratory exams, were unremarkable. Patient BMI was within normal range. Vital signs, including peripheral oxygen saturation, were within normal limits. Prior to this he had well‐controlled hypertension for years on lisinopril and propranolol, along with dyslipidemia treated with atorvastatin and ezetimibe. Family history was unremarkable. The patient previously worked as a postman, remained physically active, and did not consume tobacco or alcohol.

In terms of work up for erythrocytosis, no clinical features suggested hemoconcentration, sleep apnea, renovascular disease, or apparent cardiopulmonary disease. However, as part of his workup for refractory hypertension, the patient underwent an electrocardiogram (ECG) and transthoracic echocardiogram (TTE). While no abnormalities were detected on ECG, a large mass adherent to the RV free wall was seen on TTE. A subsequent transesophageal echocardiography and cardiac magnetic resonance imaging (MRI) corroborated the findings and demonstrated a large heterogenous mass adherent to the RV free wall and posterior leaflet of the tricuspid valve. No extension was noted outside the RV chamber, and views of the pericardium and IVC showed no involvement. The RV possessed severely reduced systolic function of 17% with no outflow obstruction. Left ventricular size was normal with an ejection fraction of 54%.

**FIGURE 1:**
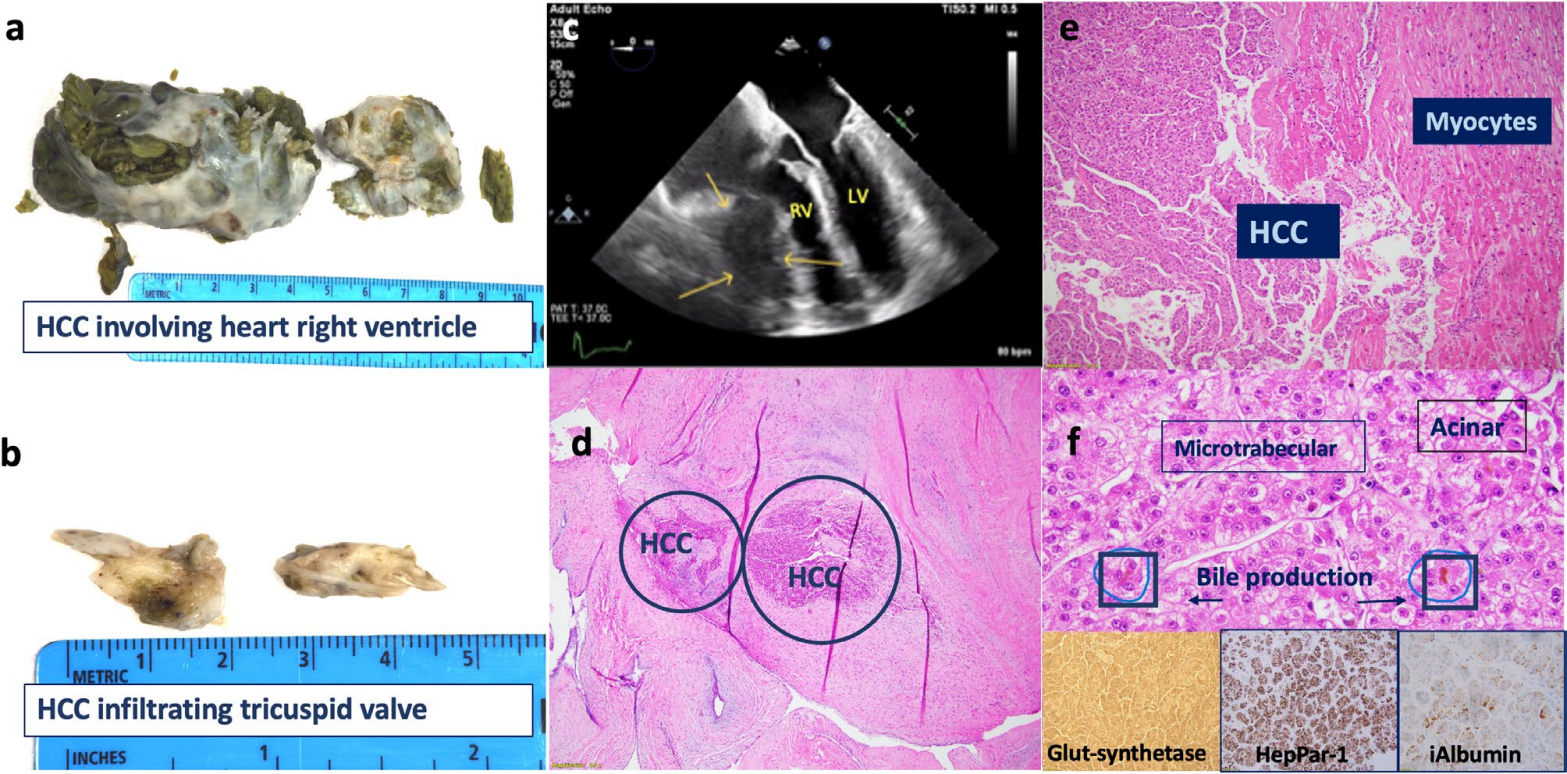
Gross and histological appearance of resected RV and tricuspid valve metastatic HCC. (a) HCC metastasis to RV. (b) HCC infiltrating tricuspid valve. (c) Transesophageal echocardiogram demonstrating mass adherent to right ventricle, RV; left ventricle, LV. (d) H&E stain of HCC RV metastasis. (e) Magnified H&E stain of HCC RV metastasis demonstrating myocyte involvement. (f) Features of CTNNB1/β‐catenin mutated HCC, demonstrating microtrabecular, acinar, and bile production with positive staining of glutamine‐synthetase, hepatocyte paraffin‐1, and iAlbumin.

A plan was made for surgical resection given the concern for metastasis and RV dysfunction. In the following months leading up to surgery, the patient started experiencing right‐sided chest pain, dizziness, and dyspnea on exertion. No further work up was done to ascertain the cause of polycythemia, as it was assumed to be secondary to the tumor. Therapeutic phlebotomy was commenced to treat worsening erythrocytosis, with Hb peaking at 17.9 g/dL, despite treatment. The patient subsequently underwent resection of RV mass with tricuspid valve replacement 6 months after his initial presentation. Multiple tan‐green, soft to firm fragments of tissue measuring 7.5 × 3.7 × 2.2 cm and weighing 54 g were obtained. The diagnosis of metastatic HCC was made based on characteristic histopathological findings, which included microtrabecular and acinar patterns with bile staining. Immunohistochemistry was also performed for confirmation, with tumor cells positive for hepatocyte paraffin‐1, glutamine synthetase, glypican‐3, arginase‐1, iAlbumin, and CD10 (Figure 1). Following successful surgery, the patient was discharged within one week and was scheduled for oncology follow up for further management. No recurrence of metastasis or polycythemia was seen 1 month following surgery.

## 3 Discussion

Cardiac metastasis of HCC is a clinically uncommon presentation, with diagnosis being difficult and often delayed by non‐specific symptoms, inadequate imaging, and low clinical suspicion [4, 5, 6]. Isolated HCC metastasis without IVC involvement is often a result of hematogenous rather than direct or transvenous spread [3]. Although a few previous reports have described isolated recurrence of HCC following primary resection, no cases of HCC cardiac metastasis presenting with hypertension and erythrocytosis have been reported before in the literature [3, 4]. In a retrospective study of reported symptoms associated with HCC cardiac metastasis, most patients were asymptomatic (39.5%), while bilateral lower leg edema (37.5%) and exertional dyspnea were the second and third most common symptoms, respectively [6]. Other uncommon presentations such as pericardial disease, abnormal murmurs, and ECG changes have also been reported in the literature [4, 6]. This case posed numerous features that proved a diagnostic challenge. The initial presentation of erythrocytosis and refractory hypertension developed gradually during the surveillance period. The patient's history of longstanding primary essential hypertension and frequent blood donations prior to the initial HCC diagnosis also lowered suspicion for disease recurrence. This was compounded by absent findings on surveillance CT scans and normal AFP levels. Previous case reports and studies have reported associations between HCC and hypertension, likely from abnormalities in the renin‐angiotensin system [7]. However, in this case, erythrocytosis may have contributed to the development of hypertension. Possible explanations for secondary polycythemia include tumor paraneoplastic production of erythropoietin, hypoxia‐driven response to tumor invasion, and cardiac dysfunction itself. While no work up was done for primary polycythemia, the absence of erythrocytosis after resection of the mass suggests a secondary cause.

Besides the atypical presentation, the clinicopathological features of the metastatic tumor offer valuable insight to the case. While the primary tumor was not available for comparison, histopathological features of the metastatic tumor include micro‐trabecular and acinar patterns, moderate differentiation, bile production, and glutamine synthetase expression. These features are highly characteristic of the non‐proliferative HCC phenotype, associated with CTNNB1 gene mutations involved in the Wnt/β‐catenin pathway. In addition, this histopathological pattern is also associated with the development of HCC in the absence of classic risk factors and low serum AFP levels [8, 9]. The direct exposure of cardiac myocytes to bile acids may have contributed to the severe RV dysfunction seen, as excess bile acid exposure in cardiomyocytes can lead to apoptosis, arrhythmogenicity, and metabolic dysfunction [10].

In general, the prognosis for HCC cardiac metastasis is poor, with a reported overall survival rate of 32.5% [5]. A review by Zhang et al. of 13 cases of isolated HCC RV metastasis reported survival times ranging from immediate post‐surgery to 9 months. In addition, most cases were diagnosed initially by TTE and treated surgically [4]. While TTE is a commonly utilized image modality for initial diagnosis, cardiac MRI is considered the gold standard for evaluating cardiac metastases due to its ability to characterize soft tissue and surrounding anatomy [4]. Treatment options for HCC cardiac metastasis include surgical removal, chemotherapy, targeted therapy, and radiotherapy [3, 4, 5, 6]. While no specific therapy has been shown to be exclusively effective against cardiac HCC metastasis, surgical resection is often performed for symptom alleviation, confirmation of diagnosis, and possible survival benefit [4, 5].

## Ethics Statement

The authors declare that the study was conducted in line with the principles of the Declaration of Helsinki. In addition, diligent care has been taken to ensure that the International Committee of Medical Journal Editors (ICMJE) recommendations have been adhered to with respect to preserving the patient's privacy and identity. Institutional Review Board (IRB) approval was not required according to the University of Pittsburgh Medical Center IRB review board policy.

## Consent

A written informed consent was obtained from the patient and is available for review by the Editor‐in‐Chief of this journal. A written informed consent was obtained from the patient for the publication of data related to this article, including images, photographs, and other clinical information.

## Conflicts of Interest

The authors declare no conflicts of interest.

## Data Availability

All accessed data and literature relevant to this study are included in this article.
